# Host Genetics and Microbiota Interactions in Colorectal Cancer: Shared or Independent Risk?

**DOI:** 10.3390/microorganisms10112129

**Published:** 2022-10-27

**Authors:** Irati Romero-Garmendia, Koldo Garcia-Etxebarria

**Affiliations:** 1 Department of Genetics, Physical Anthropology and Animal Physiology, University of the Basque Country (Universidad del País Vasco/Euskal Herriko Unibertsitatea), 48940 Leioa, Spain; 2 Gastrointestinal Genetics Group, Biodonostia, 20014 San Sebastián, Spain; 3 Centro de Investigación Biomédica en Red de Enfermedades Hepáticas y Digestivas (CIBERehd), 08036 Barcelona, Spain

**Keywords:** colorectal cancer, genetics, genomics, microbiota

## Abstract

The role of microbiota in colorectal cancer has been studied since alterations in its composition were observed. In addition, there are more and more pieces of evidence that microbiota could be implicated in colorectal cancer progression. Thus, the components of the microbiota could be biomarkers for the diagnosis and prognosis of colorectal cancer. In addition, it is important to address how the microbiota interacts with the host and how the host shapes the microbiota, in order to understand the biological pathways and mechanisms involved in their relationship and the consequences of their interactions in colorectal cancer. Thereby, it could be possible to find feasible measures and treatments to prevent or better diagnose colorectal cancer. In this review, we will try to summarize the role of the microbiota in colorectal cancer and its interactions with the host and the host genetics, coming to some conclusions that could be useful to find the gaps in our knowledge and propose future steps in this field.

## 1. Introduction

Colorectal cancer (CRC) causes a high burden in developed countries and is a major concern for health systems. Worldwide, around one out of ten diagnosed cancers are CRC and, among cancers, the second cause of death [1,2]. In Europe alone, each year 500,000 cases of CRC are diagnosed and 242,000 persons die [3]. In addition, CRC is the third most diagnosed cancer among males and the second leading cause of death; among females, it is the second most diagnosed cancer and the third leading cause of death [3]. This trend is observed in the majority of European countries [3]. Thus, screening strategies have been used for its early detection, since the success of the treatment is conditioned by the stage where it has been diagnosed; and biomarkers for diagnosis, prognosis, and success of treatment of CRC have been widely searched for [4]. In order to find feasible biomarkers, it is paramount to understand the biological factors involved in the risk of CRC and its etiology.

Although CRC can be inherited, the majority of cases are sporadic; therefore, CRC is a heterogeneous disease. Among the inherited syndromes, the two main subtypes are Lynch syndrome (the 2–4% of diagnosed CRCs) and polyposis syndromes (e.g., adenomatous polyps; Peutz–Jeghers polyps or serrated polyps) [5]. In the case of sporadic tumors, they can be classified as hypermutated cancers with microsatellite instability and nonhypermutated cancers with copy number alterations [6]. Due to the heterogeneity of CRC, different methods have been proposed to classify such tumors [6]. For example, Consensus Molecular Subtypes proposed four types of tumors [6]: hypermutated and immune tumors (14% of CRCs), canonical tumors (37%), metabolic tumors (13%), and mesenchymal tumors (23%), while 13% of tumors show mixed features.

There are two main ways to develop sporadic CRC [7]. In the conventional way, the precursor lesions are conventional adenomas (which showed tubular, tubulovillous, or villous histology) that progress to CRC [7]. In the serrated way, however, the precursor lesions are serrated adenomas (which showed stellate architecture of the crypt epithelium) [7]. Around two-thirds of sporadic CRC are developed in the conventional way, while the serrated way occurs in the other third of the CRC cases [7].

Moreover, CRC can be developed as a consequence of inflammatory processes [8,9]. More specifically, it has been observed that patients with inflammatory bowel disease have a greater risk of develop CRC [10]. Lifestyle and environmental factors are other sources of risk for CRC, since physical activity and diet can influence its development [11]. In addition, the role of microbiota in CRC has been studied and proposed as a risk factor and possible biomarker [9,12].

In this review, we will try to summarize the current understanding of the role of microbiota, highlighting the interactions between host genetics and microbiota in CRC risk. First, we will discuss the role of the microbiota in CRC and its progression, both the taxa that are involved and the putative biological pathways involved; then, we will review how the genetics of the host and microbiota interact; then, we will finish with some conclusions and by proposing some possible steps for the future.

## 2. Microbiota Involvement in CRC

### 2.1. Microbiota Composition

When microbiota is analyzed, bacterial composition is usually analyzed. The studies about the role of archaea, viruses, fungi, and parasites are scarcer, and their effect on the rest of the microbiota is less known. However, there are valuable studies that have contributed to the understanding of the diversity in the composition of the microbiota and the complex interactions between the different members of the microbiota.

#### 2.1.1. Bacteria

There is no doubt that bacteria have been widely analyzed in order to study their role in CRC. Both metagenomics and candidate-based analyses have been used to identify potential bacteria involved in CRC and its progression. In those studies, among other species, some bacterial species have been proposed as important players in CRC. For example, it has been observed that the abundance of *Fusobacterium nucleatum* is higher in CRC and, therefore, it is a possible biomarker of CRC [13]. In addition, it has been linked to oncogenic and inflammatory processes [14,15,16], and its abundance is affected by diet, especially by fiber consumption [17]. Another species associated with CRC is *Bacteroides fragilis*, especially its enterotoxigenic strains [18], and patients infected with *B. fragilis* have more risk of developing CRC [19]. In animal and cellular models, it has been observed that *B. fragilis* activates IL-17-dependent STAT3 and NF-κB pathways, leading to carcinogenesis [20,21] and damage to colonic cells [21]. Interestingly, the nontoxigenic *B. fragilis* strains could have protective effects [22,23]. In the case of *Eschericiha coli*^pks+^, it could be implicated in CRC since its genotoxins could damage the DNA of host cells [24,25], it induces the apoptosis of host cells [26], and it could cause chromosomal aberrances and genomic insatiability in colon cells [27].

The scope of this review is not to summarize all the studies about microbiota involvement in CRC. Instead, we have selected some examples to show the complexity of studying the composition of bacteria, and the differences present between studies, between the stages of CRC progression, and between the locations of CRC. In those studies, the mentioned bacteria and a lot of other taxa have been implicated in the development of CRC and are proposed as biomarkers.

In a study where stool samples from adenoma, CRC, and healthy patients were analyzed, the CRC microbiota composition (Bray-Curtis distance) was different between adenoma and healthy samples [28]. In addition, CRC samples showed richer composition than adenoma and healthy samples, while the Shannon index was not different between the three groups [28]. Regarding the differences in the abundance of taxa, Bacteroidetes, Firmicutes, and Fusobacteria phyla differed in abundancy between CRC and healthy samples, while Bacteroidetes and Firmicutes were significantly different between adenoma and healthy samples [28]. In addition, in CRC samples *Fusobacterium*, *Staphylococcus*, and *Parvimonas* were more abundant, while *Coprococcus*, *Blautia*, *Clostridium*, and *Streptococcus* were depleted [28]. Comparing adenoma and CRC, *Staphylococcus*, *Parvimonas*, and *Adlercreutzia* were more abundant in CRC, and *Coprococcus*, *Blautia*, and *Dorea* were more abundant in adenoma [28]. Moreover, the abundance of *Bulleidia*, *Fusobacterium*, *Butyrivibrio*, *Peptostreptococcus*, *Staphylococcus*, *Parvimonas* and *Selenomonas* increased with the stage of CRC, while Lachnospiraceae showed the inverse trend [28].

Analyzing whole-genome sequencing data from fecal samples, where samples from advanced-stage CRC patients and healthy controls were compared, CRC patients showed higher diversity (in species richness, Shannon index, and Simpson index) [29]. In addition, 10 species (*Parvimonas micra*, *Peptostreptococcus stomatis*, *Gemella morbillorum*, *Fusobacterium nucleatum*, *Streptococcus anginosus*, *Dialister pneumosintes*, *Peptostreptococcus anaerobius*, *Streptococcus* sp. KCOM 2412, *Ruminococcus torques*, and *Filifactor alocis*) showed higher abundance in CRC, while 174 bacterial species from 84 genera (mainly from *Enterobacter*, *Klebsiella*, *Streptococcus*, *Lactobacillus*, *Citrobacter*, *Bifidobacterium*, *Bacteroides*, and *Clostridium* genera) demonstrated less abundance [29].

In another study, stool samples of CRC patients *Fusobacterium*, *Porphyromonas*, *Pseudomonas*, *Streptococcus*, and *Gemella* were more abundant in the stool of healthy controls, while Ruminococcaceae and Lachnospiraceae were depleted [30]. In addition, in the same study, publicly available datasets were analyzed. It was observed that bacterial taxa were associated with the CRC stage [30]: the abundance of *Bacteriodes* was low in all stages, while the abundance of *Gemella* was higher in stages I, II and IV; the abundance of *Campylobacter* was higher in stages II and IV, with *Fusobacterium* more represented in stage II. In addition, the abundance of *Fusobacterium* was higher when compared with adjacent healthy tissue; its abundance was also higher in the CRC subtypes: hypermutated and immune tumors, and metabolic tumors [30].

Moreover, there are differences in microbiota composition between left and right colon cancers.

One work compared the fecal microbiota of patients with tumors located in the left colon and right colon [31]. They did not observe differences in alpha diversity (OTU richness, Shannon index, and Pielou’s evenness), but beta diversity (Bray-Curtis distance) was higher in left colon tumors [31]. In addition, the abundance of Fusobacteriota was higher in left colon cancer, while Micrococcaceae was higher in right colon cancer [31]. Additional analyses showed that *Blautia*, Eryspelotrichales, *Holdemanella*, *Faecalibacterium*, *Subdoligranulum*, and *Dorea* were more abundant in right colon cancer, while *Fusobacterium* was more abundant in left colon cancer [31].

Another work compared the microbiota composition of tumors and adjacent tissue, both in left colon cancer and right colon cancer [32]. Interestingly, the microbial composition of healthy tissue was significantly different between the left and right colon in terms of bacterial abundance, species richness, and bacterial diversity, while the tissues from tumors did not show that difference either in alpha or beta diversity [32]. Thus, although in normal conditions the microbiota present in the left and right colon differ, the presence of tumors homogenizes the microbiota of both sides [32]. In addition, Methylophilaceae and Vadin BE97 families and *Alloprevotella*, *Intestinibacter*, *Rombutsia*, and *Ruminococcus* genera were more abundant in tumors of left colon cancer, while in right colon cancer was not any significant more abundant taxa [32]. When healthy tissues and tumors were compared, in the left colon 22 taxa had a different abundance (Cyanobacteria phylum; Melainabacteria class; Corynebacteriales, and Gastranaerophilales orders; *Clostridiales vadin* BB60, Corynebacteriaceae, Dietziaceae, Eggerthellaceae, Rikenellaceae, Ruminococcaceae families; *Acidaminococcus*, *Alistipes*, *Aquabacteriaum*, *Dietzia*, *Eubacterium*, *Paraprevotella*, *Prevotella* 9, *Lachnoclostridium*, *Lachnospira*, *Paeniclostridium*, *Ruminococcus*, and *Subdoligranulum* genera), while in the right colon only 3 taxa showed differential abundance (Porphyromonodaceae family, and *Lachnospira* and *Porphyromonas* genera) [32].

#### 2.1.2. Archaea

The abundance of archaea compared to bacteria is lower, and next-generation sequencing techniques have to be used to capture its composition since it is difficult to culture them [33]. In the case of CRC, and using fecal samples, changes in alpha diversity (invsimpson and richness indices) were not detected but the composition between early-stage CRC, advanced-stage CRC, and healthy controls was different [33]: CRC patients showed a higher abundance of halophilic archaea (especially species from *Halopelagius*) and lower abundance of methanogens archaea (*Methanosphaera*, *Methanococcoides*, *Methanocorpusculum*, *Methanocaldococcus*, and *Methanobacterium*). In addition, early-stage CRC patients showed a higher abundance of *Methanocaldococcus* and *Methanotorris* than patients with advanced CRC [33]. In the case of adenoma, the halophilic archaea *Halohasta*, *Haloferax mediterranei*, *H sediminicola*, and *Natrinema J7-2* were more abundant in patients with adenoma compared to healthy patients [33]. Using 28 species of the mentioned taxa, it could be possible to use them as biomarkers to correctly classify patients with adenoma and CRC [33].

Regarding the interactions between archaea and bacteria, while the diversity of archaea and bacteria was correlated in healthy controls and patients with adenoma, there was no correlation in CRC patients [33]. Thus, the relationship between commensal archaea and bacteria seems disturbed in CRC [33]. In fact, there was a positive association between bacteria enriched in CRC (*Bacteroides fragilis*, *Bacteroides caccae*, *Oscillibacter PEA192*, *Lachnoclostridium YL32*, *Clostridium bolteae*, and *Alistipes shahii*) and the previously mentioned archaea enriched in CRC, as well as between *H. aranensis*, *Haloplanus CBA1113* and *B. fragilis* [33]. In addition, the archaea present in CRC were negatively associated with butyrate-producing *Clostridium beijerinckii* and *Clostridium kluyveri* bacteria. That is to say, there could be an antagonistic relationship between archaea halophiles and gut-protective bacteria [33]. This antagonistic relationship was also observed in adenoma patients [33].

#### 2.1.3. Viruses

In the case of the virome, various studies carried out showed that it is also altered in CRC.

In fecal samples of CRC patients, there is an increase in the number of bacteriophages and their diversity compared with healthy samples [34]. This increased diversity in bacteriophages was correlated with a reduction in bacterial diversity in CRC samples, and the diversity of viruses and bacteria was negatively associated with healthy samples, but not in CRC [34]. In addition, the abundance of 22 viral genera was enough to differentiate CRC from healthy samples [34]: *Orthobunyavirus* was the viral taxon with more weight to discriminate both groups, and viral taxa such as *Tunalikevirus*, *Phikzlikevirus*, *Betabaculovirus*, *Sp6likevirus*, *Sfi21dtunalikevirus*, *Punalikevirus*, *Lambdalikevirus*, *C2likevirus*, and *Mulikevirus*, showed different abundances between CRC and healthy samples. That ability was validated in different cohorts, but those viral taxa were not able to differentiate adenoma from healthy samples [34]. In addition, there were differences in the viral composition between early stages of CRC and advanced stages of CRC. In addition, viral composition could be associated with CRC prognosis [34].

Moreover, as mentioned previously, the relationships between bacteriophages and bacteria were disrupted in CRC [34]. The analyses showed that the bacteriophages *Streptococcus* phage SpSL1, *Streptococcus* phage 5093, *Vibrio* phage pYD38-A, *Enterobacteria* phage HK544, *Streptococcus* phage K13, *Parabacteroides* phage YZ-2015b, *Nocardia* phage NBR1, *Erwinia* phage phiEaH2, *Enterobacteria* phage HK544 and *Streptococcus* phage 5093 changed their connections with microbial taxa in early stages of CRC and/or advanced stages of CRC [34].

Another study analyzed the virome in fecal samples of adenoma, CRC, and healthy patients [35]. In this case, there was not any difference in alpha or beta diversity (Shannon diversity, richness, and Bray-Curtis metrics) [35]. However, the use of viral taxa was able to discriminate between groups, performing almost as well as bacterial taxa [35]. The viral taxa with more weight were various phages of *Siphoviridae* and *Myoviridae* bacteria [35]. In addition, the viral taxa could be useful to differentiate the health–adenoma–CRC progression, since their abundance was different in each stage [35]. Interestingly, the phages that drive the progression were temperate (they integrated into bacteria genome to form lysogeny and then transit to lytic mode), and the presence of nonbacterial viruses was scarce [35]. In this study, there was a low correlation between phage and bacterial abundance but the most relevant phages in CRC had a broad bacterial host range [35].

In another study of fecal samples of CRC and healthy patients of three cohorts, the alpha diversity of bacteriophages was higher in CRC, and beta diversity showed that the bacteriophages of CRC patients and healthy patients were different [36]. The number of bacteriophages that showed a different abundance in each cohort was different, but, interestingly, five phages were common to the three cohorts [36]: *Peptacetobacter hiranonis* phage, *Fusobacterium nucleatum animalis* 7_1 phage, *Fusobacterium nucleatum polymorphum* phage, *Fusobacterium nucleatum animalis* 4_8 phage, and *Parvimonas micra* phage. In addition, those five phages were validated in additional two cohorts with good accuracy [36].

It must be pointed out that the viruses that infect the host have been proposed as a risk factor in CRC. The role of human papillomavirus in CRC has been studied and, although there are conflicting results, it could be a risk factor in a group of CRCs [37]. In addition, the role of human polyomaviruses and human herpesviruses in CRC has been studied, but there were no clear results [38].

#### 2.1.4. Fungi

The studies that have analyzed the composition of the mycobiota showed that there are alterations in fungal taxa in CRC.

In a study analyzing the mycobiota of fecal samples, different composition of fungal taxa between CRC patients and healthy controls was detected [39]. In addition, early-stage CRC and advanced-stage CRC showed different fungal profiles [39]. However, the alpha diversity (species richness and Shannon diversity) was not different between CRC and healthy samples [39]. Regarding fungal taxa, differences in Basidiomycota:Ascomycota ratio were observed in CRC samples; and a higher abundance of Malasseziomycetes class; Pisolithaceae, Marasmiaceae, Malasseziaceae, Erysiphaceae, Pseudorotiaceae and Chaetomicaeae families; and *Malassezia*, *Moniliophthtora*, *Rhodotorula*, *Acremonium*, *Thielaviopsis*, and *Pisolithus* genera were detected in CRC, while the Saccharomycetes and Pneumocystidomycetes classes; and Lipomycetaceae families were depleted [39]. In addition, it was concluded that *Aspergillus flavus*, *Kwoniella mangrovensis*, *Pseudogymnoascus* sp. VKM F-4518, *Debaryomyces fabryi*, *A. sydowii*, *Moniliophthora perniciosa*, *K. heavenensis*, *A. ochraceoroseus*, *Talaromyces islandicus*, *Malassezia globosa*, *Pseudogymnoascus* sp. VKM F-4520, *A. rambellii*, *Pneumocystis murina*, and *Nosemia apis* species could be used to discriminate between CRC and healthy samples [39]. Those taxa were further validated in independent cohorts, showing a good performance in differentiating CRC and control samples, but did not perform well in differentiating adenomas from healthy controls [39].

Moreover, the correlation between fungal phyla was different in CRC and healthy samples: in CRC there were correlations between Ascomycota, Basidiomycota, and Mucoromycota that were not detected in healthy samples [39]. Those changes were also detected in the correlations between fungi and bacteria: in CRC there were more correlations and co-exclusive relationships than in healthy samples [39]. Fungal taxa Pucciniomycetes, Exobasidiomycetes, and Malasseziomycetes showed co-exclusive relationships with bacterial taxa Alphaproteobacteria, Gammaproteobacteria, Deltaproteobacteria, and Cytophagia in CRC that were not present in healthy samples [39]. In addition, the relationships of Leotimycetes, Sodariomycetes, and Eurotiomycetes fungal classes become co-exclusive in CRC. As a consequence of the mentioned changes, the homeostasis between bacteria and fungi in CRC was disrupted, and the changes in bacterial composition could lead to changes in fungal composition [39].

In another study, mycobiota from adenoma, CRC, and healthy fecal samples were analyzed [40]. Regarding ecological features, the number of OTUs, Chao 1 index, and Shannon index was not different between groups, while the Simpson index was different between adenoma and healthy groups [40]. The fungal taxa *Glomus*, *Microascaceae* spp., *Pseudolagarobasidium*, *Pseudozyma*, and *Sordariaceae* spp. showed different abundancies among the three groups [40]. In addition, *Trichosporon*, Sordariales, *Mucor*, and *Sordariales* spp. were the taxa more characteristic of CRC; *Sordariomycetes* of adenomas; and Lasiosphaeriaceae and *Cladorrhinum* of healthy samples [40]. In the case of CRC stage, advanced-stage CRC showed more diversity than early-stage CRC; and Microbotryomycetes, Sordariomycetes, Microascaceae, *Sordariales* spp., *Lasiosphaeriaceae* spp., *Sordariales* spp., and Microascales were more abundant in advanced-stage CRC, while Pleosporaceae and *Alternaria* were more abundant in early-stage CRC [40]. When adenoma and CRC were compared, there was no significant difference [40]. Moreover, the relationships between fungal taxa were different between CRC and healthy controls, and adenomas and CRC [40].

It is worthy of not that, when the mycobiota of adenomas and adjacent tissues was compared, less diversity was detected in adenomas [41]. In addition, Basidiomycota was less abundant in adenomas, Spizellomycetales order was more abundant in adenomas, and Paraglomerales was more abundant in nonadvanced adenomas [41].

#### 2.1.5. Parasites

In the case of eukaryotic parasites, their presence has been observed in CRC. For example, the presence of *Cryptosporidium*, a protozoan that causes diarrhea, was detected in CRC patients [42,43]. In addition, in another cohort, the presence of *Cryptosporidium* was higher in CRC patients than in patients with persistent gastrointestinal symptoms and in healthy populations, while *Cryptosporidium hominis* and *Cryptosporidium parvum* were identified in CRC samples [44]. In another study, where its presence was higher in CRC patients, it was concluded that *C. parvum* was the species associated with CRC [45]. Another parasite detected in CRC patients is *Pentatrichomas hominis*, whose presence was higher in CRC patients than in healthy populations [46].

Moreover, one study showed that in the presence of *P. hominis*, the composition of the microbiota was different in CRC compared with CRC patients without a *P. hominis* infection [47]. In the presence of *P. hominis*, the abundance of *Ruminococcaceae* UCG-002 and *Eubacterium eligens* was lower than in its absence, and the abundance of *Flavonifractor* spp., *Lachnoclostridium* spp., and *Ruminococcus gnavus* was higher [47].

In summary, the composition of microbiota in CRC changes, but there are conflicting results (Table 1). Although it is clear that the composition changes, the differences in the diversity are not clear. The higher diversity in CRC could be a consequence of the presence of harmful bacteria or bacteria better adapted to the changes in the tumor microenvironment [28,48]. However, in some studies there were not any differences in the diversity. In fact, this discrepancy is observed in bacteria, viruses, and fungi. In addition, although few taxa seem to be consistently altered (e.g., *Fusobacterium*), the taxa altered in each study was different; and the similarities between adenoma and healthy samples are higher than with CRC. Thus, it is more complicated to diagnose adenoma using only microbiota data. It must highlighted that the differences between CRC stages and location, and the changes in the relationship between the components of microbiota, could be useful as biomarkers and to find new biological mechanisms involved in CRC.

However, the variability in the taxa that are involved in the changes could suggest that there are factors shaping the microbiota that are different between the studies or the population studied. In addition, the biases introduced by the methods used cannot be ruled out. One study showed that 16s rRNA sequencing is prone to biases, since the results could change depending on the DNA extraction method and analytical pipeline used [49]. This effect was lower in abundant and well-characterized genera; therefore, the analysis of low-frequency genera is more limited and prone to bias [49]. Thus, the lack of consistency between studies could be a methodological effect rather than a biological effect, and that could be a limitation in efforts to identify all genera and species involved in CRC development and progression.

### 2.2. Pathways and Biological Functions of the Microbiota

One of the most remarkable features of bacteria is the plasticity of their genomes. Thus, it is not enough to know what taxa are present in CRC, and it is necessary to know the biological functions that they can carry out. There have been technical limitations to studying the gene content of microbiota, but its study is increasingly feasible.

However, it must pointed out that microbiota is functionally redundant in healthy persons [50,51]. Although a lot of taxa carrying diverse genes can be found in the microbiota, those genes have similar functions [50,51]. That is, the composition of microbiota varies between individuals, but the biological functions present in the microbiota are conserved. Thus, it is necessary to elucidate if this functional redundancy is perturbed in CRC, and how. To study the bacterial genes present, they can either be indirectly inferred using the microbiota composition, or directly by whole-genome sequencing of bacterial content.

In a study where stools from adenoma, CRC, and healthy patients were analyzed, based on the bacterial content, the biological functions were inferred [28]. In this case, the pathways related to amino acids biosynthesis, fermentation (isobutanol, acetate, and lactate), glucose metabolism (gluconeogenesis, glycolysis, and glycogen biosynthesis and degradation), saturated fatty acids, reductive incomplete TCA, nitrate degradation and methanogenesis were different in CRC samples [28].

Moreover, when the left and right colon cancer were compared, since the microbiota had a different composition, the microbial pathways present were different [31]. In the case of left colon cancer, “L-lysine fermentation” and “cob(II)yrinate a,c-diamide biosynthesis I” pathways were predominant, while there was no significantly enriched function in right colon cancer [31].

One study analyzed the bacterial gene content of CRC and healthy stools [52]. There were no significant differences in KEGG pathways between the genes present in CRC and healthy stools, but two KEGG modules (leucine degradation and guanine nucleotide biosynthesis) were significantly enriched [52]. In addition, 12 orthologous gene groups were more abundant in CRC (e.g., “Glycine reductase”, “N-acetylornithine carbamoyltransferase”, and “Rrf2 family transcriptional regulator”), while 7 were less abundant (e.g., “Type IV pilus assembly protein PilF”, “Protein-serine/threonine kinase” and “Phosphatidylglycerophosphatase B”) [52].

In another work, the gene content from whole-genome sequencing data was assigned to protein families or domains, and some differences were found between CRC and healthy samples [29]. In the case of protein domains, 7 were more abundant in CRC samples, and those domains were related to bacterial invasion and adhesins; while 5 were reduced domains related to antibiotic resistance, bacteriophage maturation, and threonine biosynthesis [29]. In the case of protein families, one family was more abundant in CRC (associated with proline iminopeptidase) and the other less abundant (associated with threonine biosynthesis) [29].

Moreover, novel methods have been developed to cluster bacterial genes using their co-abundance and, therefore, to find consistently disturbed genes in CRC [53]. That approach was used to analyze publicly available datasets and in healthy samples, the largest co-abundant gene group was from *Clostridia*, while in CRC the origin of those genes was taxonomically more diverse [53]. In addition, the largest co-abundant genes cluster in CRC had genes involved in the “metabolism of glycine/sarcosine/betaine”, “protection from the biochemical damage induced by reactive oxygen species”, “uptake of p-aminobenzoyl-glutamate”, and “transporters responsible for importing iron”, biological functions that are related to cancer progression [53].

To sum up, the studies where the biological capabilities of bacteria are analyzed are fewer than the ones analyzing the content, and few pathways or biological functions have been detected to be different in CRC. In accordance with the functional redundancy of microbiota, although the taxa present can change, the main functions of microbiota seem to be conserved and few pathways related to cancer progression can be detected.

## 3. Interactions between Host Genetics and Microbiota in CRC

We have recapitulated some of the changes that have been observed, both in composition and biological functions in microbiota. However, is the host somewhat driving those changes, or does the microbiota change the host cells?

How host genetics shapes microbiota composition and microbiota the biological functions of the host have been intriguing questions. Over the years, their study has evolved, and it has not been until recently that we have had a more precise picture of the genetic mechanisms that shape the host–microbiota interactions. The development of *omics* approaches has facilitated the analysis and inference of correlations and interactions between the host genetics (and its different layers) and microbiota. Although the number of such studies is scarce, the results of those studies have highlighted that there are some mechanisms shared by host genetics and microbiota, and other mechanisms that are independent.

### 3.1. Shared Risk Components

#### 3.1.1. Genetic Variation

The study of the genetic variants of the host that affect the abundance of bacterial taxa has been a valuable asset to get genetic signatures related to microbiota composition [54]. Based on those genetic signatures of microbiota composition, it has been analyzed the contribution of various phyla to CRC risk using Mendelian Randomization analyses [55]. Although it is an indirect method, it gives information to determine if there are shared genetic mechanisms to CRC risk and the abundance of a given phylum. Although the results were weak, the genetic variants associated with the abundance of Firmicutes and Cyanobacteria phyla were also associated with the genetic risk of CRC, specifically with the genetic risk of left colon cancer [55]. It must pointed out that in the study in question, the genetic variants involved in total cholesterol levels were determined to be a risk factor in left colon cancer [55]. Thus, it cannot be ruled out that modifiable risk factors such as cholesterol levels could shape the risk of CRC through the host genetic and/or microbiota composition.

As mentioned previously, strains of *E. coli*^pks+^ have been involved in CRC risk. The pks pathogenic island carries genes involved in colibactin, a genotoxic that could cause mutations in host cells [56]. The long-term exposure of *E. coli*^pks+^ causes a distinct mutational signature in organoids, showing a bias towards T to N substitution and a single T deletion at T homopolymers [56]. In fact, these two mutational signatures were enriched in CRC samples [56]. In addition, a study showed that the transplant of the microbiota of CRC patients to a mice model altered the DNA of the cells of the gut of the mice [57]. Thus, the microbiota could cause somatic mutations in host cells.

#### 3.1.2. Transcription

Moreover, the effect of the genes expressed by the colonic mucosa and the composition of the microbiota in CRC patients have been analyzed, among other gastrointestinal diseases [58]. In that study, they observed in CRC a correspondence between the variability of gene expression of gut tissue and microbiota composition [58]. In addition, they detected three pathways that affected the composition of the gut microbiota in the three gastrointestinal diseases that were analyzed (CRC, inflammatory bowel disease, and irritable bowel syndrome), namely “Oxidative phosphorylation”, “*RAC1* pathway” and “*ERBB1* downstream pathway”; and 52 pathways specific to CRC [58]. For example, among those specific pathways, there was the Syndecan-1 pathway [58], a pathway previously associated with tumorigenic activity and that affects the abundance of *Parvimonas* and *Bacteroides fragilis* in CRC [58], bacterial taxa that are biomarkers of CRC due to their role in the carcinogenesis. Regarding gene expression and taxa associations, the effect of the expression of 745 genes in the abundance of 120 microbes was found and those genes of the host were involved in tumor growth, progression, and metastasis [58].

Previously, we have discussed the higher presence of *Fusobacteria* in certain subtypes of CRC [30]. When the expression of genes of the host tissue was analyzed to compare the expression pattern in the presence or absence of *Fusobacteria*, pathways of the host related to inflammatory-related signaling (e.g., “IL-8 signaling” or “Th17 activation”), “aryl hydrocarbon receptor (AhR) signaling”, cellular organization, movement and invasion pathways, and metabolic pathways (e.g., cholesterol and proteoglycan metabolism) were affected [30]. In that study, the role of *Fusobacteria* in CRC was experimentally analyzed and it was observed that *Fusobacteria* has protumorigenic effects [30]. The formate produced by *Fusobacteria* activates AhR signaling, which can lead to tumor invasion, activating proinflammatory profiles [30]. That is, a bacterial metabolite affects the expression of host cells and affects CRC progression.

Moreover, a study analyzed publicly available data to determine if the composition of microbiota affected host gene expression in early-stage CRC and advanced-stage CRC [59]. The bacterial taxa with more connections with the expression of host genes in early-stage CRC were *Ilumatobacter*, *Rhodospirillum*, and *Nitrosospira*, while in advanced-stage CRC they were *Desulfurella*, *Nitriliruptor*, and *Jeotgalicoccus* [59]. Indeed, these taxa are not the usual taxa associated with CRC. Thus, this kind of novel approach is useful to unveil hidden biological mechanisms. In addition, the connected genes with these bacteria belonged to biological functions related to proliferation, biogenesis, and cell cycles in early-stage CRC, and related to migration and angiogenesis in advanced-stage CRC [59].

#### 3.1.3. Methylation

Another study analyzed the role of the microbiota in the methylation patterns of tumor suppressor genes [60], since their deregulation is one of the characteristics of CRC progression. The abundance of *Hungatella hathewayi* showed a correlation with the methylation level of tumor suppressor genes such as *SOX11*, *THBD*, *SFRP2*, *GATA5*, and *ESR1*; and *Fusobacterium nucleatum* with the methylation levels of *MTSS1*, *RBM38*, *PKD1*, and *PTPRT* [60]. In the case of the well-known tumor suppressor genes in CRC, *MLH1*, *APC*, *PTEN*, and *CDX2* showed correlations with bacterial taxa [60]. Specifically, *H. hathewayi* abundance was correlated with *CDX2*, *Streptococcus* spp. with *MLH1*, and both taxa with *APC* [60]. In addition, using an experimental approach, the upregulation of DNA methyltransferase in host cells by *F. nucleatum* and *H. hathewayi* was observed [60].

Moreover, the transplant of the microbiota of CRC patients to a mice model showed that the methylation patterns in the mice cells change and those changes were enriched in genes involved in cell growth, signal transduction, nucleic acid binding, protein synthesis, channels, and carrier proteins [57]. Then, the methylation status of several genes in samples of the original CRC patients was analyzed, and the combination of some of them was able to discriminate between CRC patients and healthy controls [57]. In addition, the samples with higher methylation changes showed a higher abundance of *Parvimonas* [57].

#### 3.1.4. Metabolites

In the case of the relationship between fecal microbiota and the metabolome, both layers were able to discriminate CRC samples from adenoma and healthy controls [28]. In addition, several genera were correlated with metabolites from the fecal microbiota [28]: Cholesteryl esters and sphingomyelins metabolites were positively correlated with the abundance of *Fusobacterium*, *Gemella*, *Parvimonas*, *Peptrostreptococcus*, and Erysipelotrichaceae, while negatively with *Coprococcus*, *Dorea* and *Blautia*. In the case of diacylphosphatidylcholines, they were negatively associated with *Coprococcus*, *Dorea*, and *Blautia*; and triacylglycerol metabolites were negatively correlated with *Desulfovibrio* and *Synergistes* genera [28]. Based on those results, a model to discriminate between adenomas, CRC, and healthy patients was developed [28].

It must be pointed out that the metabolites of the microbiota could interact with host cells’ through cell receptors. One study analyzed the metabolites present in the murine gut, and some of them were further investigated. It was observed that some metabolites derived from microbiota were able to activate pathways in human cell models [61]. This kind of interaction should be further studied in CRC, to determine if the microbiota interacts with host cells in this way and leads to the development of CRC.

#### 3.1.5. Multilevel

Although the effect of the microbiota in the fecal metabolome in adenomas and CRC was established [28], the effect of host genetics was further studied [62]. In the case of the shared risk component, it was detected that the genetic variants associated with the abundance of Bacteroidetes and Firmicutes were also involved in adenoma or CRC genetic risk [62]. In addition, genetic variants associated with cholesterol, triglycerides, phosphatidylethanolamine, and phingomyelin metabolites were associated with adenoma or CRC genetic risk [62]. There were also detected interactions between host genes of pathways related to cholesterol metabolism and the effect of genetic variants related to HDL cholesterol in adenoma and CRC risk [62]. As previously mentioned [55], the interplay between host genetic factors, host lifestyle, and microbiota could shape the risk to develop adenomas and CRC.

### 3.2. Independent Risk Components

One study analyzed the role of bacterial toxins in pre-tumorous and tumorous tissue and the effect of host genetics in CRC [63]. The results showed that the *cif* toxin gene was more present in pre-cancerous polyps or adenomas, toxins of *Escherichia coli* were more abundant in adenocarcinomas, and *E. coli*^pks+^ strains were a risk factor for CRC [63]. Interestingly, polygenic risk scores were used to measure the genetic risk of developing CRC in those patients, and there was not any significant difference between patients with pre-tumorous and tumorous lesions, and healthy individuals [63]. Thus, the host genetics did not influence the bacterial toxins and their role in the development of the lesions [63].

Previously, we have discussed some examples of shared risk components of host genetics, the fecal metabolome, and microbiota [62]. However, when the three layers were analyzed altogether using multi-*omic* integration procedures, the variance of risk to adenoma and CRC explained by each layer was different [62]: microbiota had more weight in the variance, metabolome had less weight, and the contribution of host genetics was limited. Except for the factors that explained more variance, where microbiota and metabolome showed covariance, covariance was not detected in the rest of the factors [62]. Thus, when all the information of *omic* layers is used to analyze their involvement in adenoma and CRC risk, their contribution to adenoma or CRC risk was independent [62]. In fact, the models built to predict adenoma and CRC risk based on microbiota and the metabolome [63] were more robust when information from the three layers (host genetics, microbiota, and metabolome) was used, a fact which strengthens the idea that each layer captures part of the risk to CRC. That is to say, the information is not redundant [62] (Table 2).

As we have reviewed, there are biological mechanisms that are involved in both genetic CRC risk and microbiota composition, but not all the role of microbiota in CRC is influenced by the genetics of the host. It must pointed out that the composition of the microbiota is influenced by diet [64] or lifestyle [65]. Therefore, the host could shape the composition of the microbiota by modifiable risk factors rather than genetic factors and that could partially explain that there are shared and independent risk factors. For example, the metabolism of cholesterol is one consistent pathway altered, an alteration that could partially be due to the host genetic component and another part of the diet. In addition, bacteria can shape the function of the host cell, by changing the expression and methylation patterns of key genes in CRC development.

## 4. Conclusions and Future Directions

**Figure 1:**
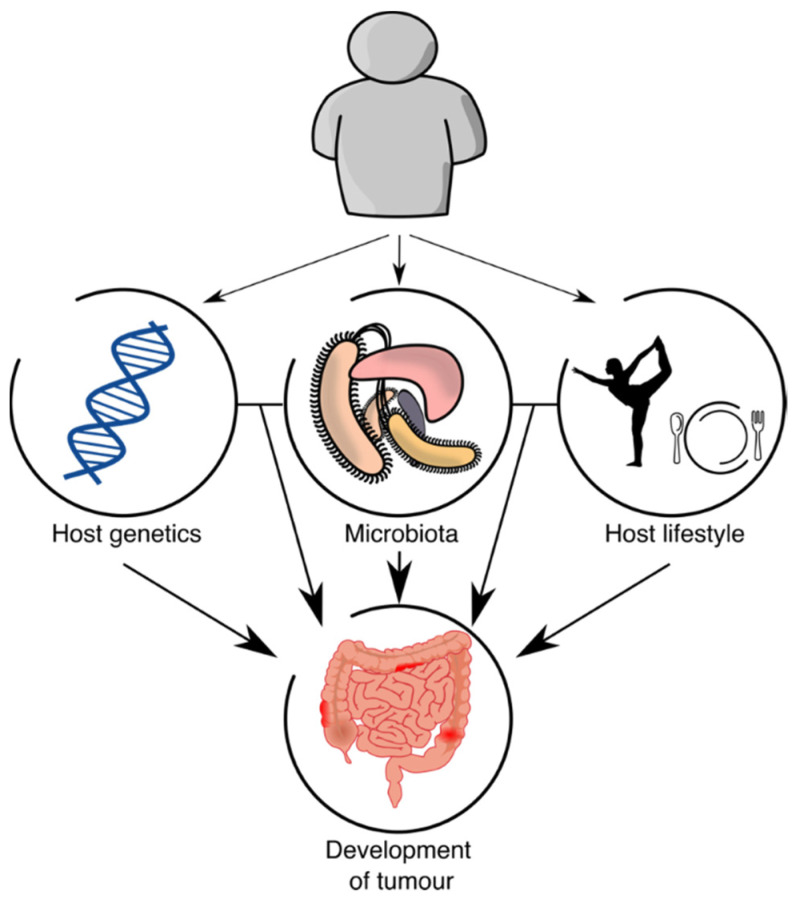
Host–microbiota interactions.

Although the interactions between host genetics and microbiota are complex and variable in CRC (Figure 1), some ideas can be concluded: there is an effect of the host on the microbiota through modifiable (lifestyle) and nonmodifiable (genetics) risk factors; the effect of those factors on CRC risk could be direct or through microbiota composition; the different members of the microbiota interact differentially in CRC; and the weight of each mechanism is different, although it seems that part of the risk is due to shared genetic factors (e.g., genetic variants of the host that affect the abundance of taxa that are involved in CRC risk), while another part is independent (e.g., the abundance of a taxon is not related to host genetics but that taxon affects host cells in CRC progression). Thus, to study CRC risk factors and to predict CRC risk probability, it seems necessary to study both components, since the information obtained by host genetics and microbiota is not redundant.

However, there is still much to understand about the interactions between host genetics and microbiota in CRC.

On the one hand, it is necessary to better understand how host genetics affect the composition of the microbiota. As we have reviewed, a lot of work has been done in this field, but more data and new methods would be necessary to properly address this issue. In fact, the use of new methods has been successful at finding hidden mechanisms and, therefore, the reanalysis of existing data applying new methods seems to be a promising approach. As a consequence, we will have better tools to measure the interactions between host genetics and microbiota in the context of CRC.

On the other hand, it is necessary to improve our studies on the microbiota itself in CRC, both in its composition and its biological functions. The development of cheaper technologies to sequence and more powerful methods to analyze the microbiota would boost this field. Specifically, the study of archaea, viruses, fungi, and eukaryotic parasites and their effect, both in the host and in bacteria composition, could boost our knowledge in this field. In addition, the study of each stage and location of the colorectal tract could improve our understanding, but we are aware of the technical and ethical limitations. Thus, the advances in the use of fecal microbiota as a proxy for the colonic and rectal microbiota would be of great value.

There is no doubt that the study of the interactions between host genetics and microbiota is a promising field that can improve our knowledge of CRC and, therefore, its prediction, prognosis, and treatment and the detection of biomarkers with clinical utility.

## Figures and Tables

**Table 1:** Summary of the findings about the components of the microbiota.

	Bacteria	Archaea	Virus	Fungi
Differences in healthy–adenoma–CRC progression				
Differences in diversity	Mixed results [28,29]	No change between CRC and healthy [33]	Mixed results [34,35,36]	Mixed results [39,40,41]
Differences in composition	Different in CRC [28,30] Few differences between adenoma and healthy [28]	Different in CRC [33]	Different in CRC [34,36] Mixed results between adenoma and healthy [34,35]	Different in CRC [39,40] Few differences between adenoma and healthy [40]
Differences in CRC stages				
Differences in diversity	More diversity in advanced-stage [29]	-	-	-
Differences in composition	Different [29,30]	Different [33]	Different [34]	Different [39,40]
Differences in CRC location				
Differences in diversity	Mixed results [31,32]	-	-	-
Differences in composition	Different but more homogenous than healthy [31,32]	-	-	-
Relationship with bacteria				
	-	Disturbed in CRC [33] Antagonistic relationships in CRC [33]	Disturbed in CRC [34]	Changes in CRC [39] Co-exclusive in CRC [39]

CRC: colorectal cancer.

**Table 2:** Summary of the findings about host–microbiota interactions in colorectal cancer.

Level	Host Affects Microbiota	Microbiota Affects Host
Genetic variation	Host variants associated with CRC could shape microbiota composition [55]	*E. coli*^pks+^ could cause somatic mutations in host cells [56]
Transcription	Host gene expression shape microbiota composition in CRC [58]	*Fusobacteria* disrupts host gene expression [30] Microbiota composition shapes host gene expression in CRC stages [59]
Methylation		*Hungatella hathewayi* and *Fusobacterium nucleatum* disrupt the methylation of tumor suppressor genes [60] Parvimonas abundance is higher in CRC patients with altered methylation [57]
Metabolites	Correlation between certain metabolites and abundance of microbiota [28]	Bacterial toxins and host genetic risk are not linked [63]
Multilevel	Host variation shapes some microbial taxa and metabolites but each layer explains part of the risk [62]	

## Data Availability

Not applicable.
